# Dynamics of ammonia oxidizers and denitrifiers in response to compost addition in black soil, Northeast China

**DOI:** 10.7717/peerj.8844

**Published:** 2020-04-21

**Authors:** Zhongzan Yang, Yupeng Guan, Ayodeji Bello, Yanxiang Wu, Jiayi Ding, Leiqi Wang, Yuqing Ren, Guangxin Chen, Wei Yang

**Affiliations:** College of Resources and Environment, Northeast Agricultural University, Harbin, Heilongjiang, China

**Keywords:** Compost addition, Ammonia oxidizer, Denitrifier, Miseq sequencing, Co-occurrence network

## Abstract

Organic fertilizer application could have an impact on the nitrogen cycle mediated by microorganisms in arable soils. However, the dynamics of soil ammonia oxidizers and denitrifiers in response to compost addition are less understood. In this study, we examined the effect of four compost application rates (0, 11.25, 22.5 and 45 t/ha) on soil ammonia oxidizers and denitrifiers at soybean seedling, flowering and mature stage in a field experiment in Northeast China. As revealed by quantitative PCR, compost addition significantly enhanced the abundance of ammonia oxidizing bacteria (AOB) at seedling stage, while the abundance of ammonia oxidizing archaea was unaffected across the growing season. The abundance of genes involved in denitrification (*nirS*, *nirK* and *nosZ*) were generally increased along with compost rate at seedling and flowering stages, but not in mature stage. The non-metric multidimensional scaling analysis revealed that moderate and high level of compost addition consistently induced shift in AOB and *nirS* containing denitrifers community composition across the growing season. Among AOB lineages, *Nitrosospira* cluster 3a gradually decreased along with the compost rate across the growing season, while *Nitrosomonas* exhibited an opposite trend. Network analysis indicated that the complexity of AOB and *nirS* containing denitrifiers network gradually increased along with the compost rate. Our findings highlighted the positive effect of compost addition on the abundance of ammonia oxidizers and denitrifiers and emphasized that compost addition play crucial roles in shaping their community compositions and co-occurrence networks in black soil of Northeast China.

## Introduction

Black soils, which are widely distributed in Northeast China, are one of the most important soil types in China (Yang et al., 2017). However, due to extensive agricultural intensification combined with the overuse of chemical fertilizers, reduction in soil fertility have occurred over the past few decades (Liu et al., 2003; Yao et al., 2017). In order to improve soil productivity, large amounts of inorganic and organic fertilizers have been frequently applied in this region (Ding et al., 2014). However, excessive and repeated input of *N* has aggravated nitrate leaching and exacerbated the emission of greenhouse gases such as N_2_O (Fowler et al., 2013; Robertson & Vitousek, 2009), with substantial effects on soil N cycling.

Soil N cycling is a complex biogeochemical process with several rate limiting steps including N-fixation, nitrification and denitrification (Kuypers, Marchant & Kartal, 2018; He et al., 2007). The distribution and functional diversity of N genes for nitrification (bacterial and archaeal *amoA*) and denitrification (*nirK*, *nirS* and *nosZ*) have previously been used to assess N-cycling functional guilds across various ecosystems (Hallin et al., 2009; Tao et al., 2018; Ai et al., 2013; Radl et al., 2015). Studies have shown that both ammonia-oxidizing bacteria (AOB) and archaea (AOA) play key roles in ammonia oxidation in agricultural soils (Jia & Conrad, 2009; He et al., 2007). However, AOA and AOB belong to different domains with different cell metabolic and biochemical process (Walker et al., 2010), they could theoretically respond differently to fertilizer application strategy (Muema, Cadisch & Rasche, 2016). Several studies demonstrated that application of organic manure or plant residues tend to increase AOA abundance and change AOA community composition, while having little effect on AOB in calcareous fluvo-aquic soils (Ai et al., 2013; Yang et al., 2018). However, others observed that organic fertilizers showed no significant effect on AOA, while AOB abundance and composition was sensitive in calcareous desert soils (Tao et al., 2017). In addition, documented publications indicated that the effect of organic application on denitrifiers can be positive (Yin et al., 2015; Cui et al., 2016; Pereg et al., 2018) or neutral (Miller et al., 2008; Sun et al., 2015) in agricultural soils. Such varying observations indicated that there is still need to examine the effect of organic fertilization on ammonia oxidizers and denitrifiers.

Alternatively, the different response of ammonia oxidizers and denitrifiers to organic amendment mentioned above was possibly due to the sampling period (Hallin et al., 2009; Tao et al., 2018; Ai et al., 2013; Radl et al., 2015). However, it should be noted that most of these studies only indicate short-term or long-term effects of organic amendment in a single sampling time (Hallin et al., 2009; Tao et al., 2018; Ai et al., 2013; Radl et al., 2015), which only capture a specific status of ammonia oxidizers or denitrifiers that may not represent the actual response. Several previous studies indicated that ammonia oxidizers and denitrifiers were subjected to noticeable temporal variations (Hussain et al., 2011; Muema, Cadisch & Rasche, 2016; Zhong et al., 2014). Therefore, a time course study is needed to analyze the evolution of ammonia oxidizers and denitrifiers under application of organic amendment.

Furthermore, both ammonia oxidizers and denitrifiers coexist in complex environment, resulting in cooperative and competitive interactions (Kuypers, Marchant & Kartal, 2018). Network analysis, which have been used recently to examine the co-occurrence of microorganisms, may reveal potential ecological roles and study the complex community organization (Deng et al., 2012). Previous studies indicated that organic input dramatically enhanced the complexity of bacterial network in agricultural soils (Ling et al., 2016; Yang et al., 2019b). However, how network patterns of soil ammonia oxidizers and denitrifiers respond to organic fertilization remained largely unknown.

In this study, we used amplicon sequencing performing using Illumina Miseq platform to provide insight into the community composition of soil N-related microbial community and quantitative PCR analysis to quantify the abundance of both ammonia oxidizers and denitrifiers. Our objectives were to (1) examine the dynamics of soil ammonia oxidizers and denitrifiers in response to compost addition during soybean growing season; (2) determine the key soil factor in shaping community compositions of soil ammonia oxidizers and *nirS* containing denitrifiers; (3) explore the co-occurrence network patterns of ammonia oxidizers and *nirS* containing denitrifiers in response to compost addition.

## Materials and Methods

### Study site and experimental design

The field trial was conducted at Xiangyang experimental farm of Northeast Agricultural University (45°45′45″ N, 126°54′46″ E), Eastern Songnen Plain, China in 2016 (Yang et al., 2019a, 2019b). The soil at this study site is classified as Mollisols. The experimental field was divided into 16 plots of 5 m × 4.5 m (2 m separating each plot) and each treatment was replicated four times in a complete randomized block design. The field has been in maize-soybean crop rotation, with chemical fertilizers applied, before 2016. Compost were applied as basal fertilizer and evenly mixed with top soil when soybean was planted. There were four treatments: (1) no compost addition (CK); (2) 11.25 t/ha compost addition (low level of compost addition, LC); (3) 22.5 t/ha compost addition (moderate level of compost addition, MC); (4) 45 t/ha compost addition (high level of compost addition, HC). The compost was produced from cattle manure and maize straw (45 days aerobic composting process). The chemical properties of the compost were: pH, 8.0; total organic carbon, 386.1 g/kg; total N, 18.4 g/kg; available P, 1.01 g/kg; NO_3_^−^-N, 0.40 g/kg; NH_4_^+^-N, 0.21 g/kg; C:N ratio, 21.0. Soybean (*Glycinemax* (L.) *Merrill*) was planted on 6th May and harvested on 29th September, 2016. No pesticide, herbicide or other chemicals were applied during the growing season. For climate and soil characteristics of the field see Yang et al. (2019a, 2019b).

### Soil sampling and soil variables

Soil sampling procedure was described in Yang et al. (2019a, 2019b). Specifically, soils were sampled on June 4 (seedling stage); July 24 (flowering stage) and 27 August (mature stage) in 2016. In each plot, five soil cores (20 cm deep, 5 cm diameter) were randomly collected and bulked together to form a single sample at each sampling time. Soil samples were then passed through 1 mm sieve to remove roots and debris, then stored at −80 °C (for DNA extraction) and 4 °C (for physicochemical analysis). Soil organic matter (SOM), total phosphorus (TP), total N (TN), available phosphorus (AP), available potassium (AK), pH and soil moisture (SM) were determined by Yang et al. (2017). Soil ammonium and nitrate were extracted with 1 M KCl solution (1:5, w/v) for 30 min and then assayed using a continuous-flow analyzer (SAN++, Skalar, Holand).

### DNA extraction and quantitative PCR

For each soil sample (48 in total), DNA was extracted from 0.25 g frozen soil samples using the PowerSoil DNA Isolation Kit (MoBio Laboratories, Inc., Carlsbad, CA, USA) according to the manufacturer’s instruction. Quantitative analysis of genes encoding catalytic enzymes of ammonia oxidation (AOA-*amoA* and AOB-*amoA*), and denitrification (*nirK*, *nirS* and *nosZ*) were performed in LightCycler^®^ 96 thermocycler (Roche Diagnostics, Indianapolis, IN, USA). The primer sets and PCR conditions of AOA-*amoA*, AOB-*amoA*, *nirS*, *nirK* and *nosZ* genes were summarized in Table S1 (Rotthauwe, Witzel & Liesack, 1997; Braker, Fesefeldt & Witzel, 1998; Throbäck et al., 2004; Francis et al., 2005; Henry et al., 2006). Amplification was conducted using the SYBR^®^ Premix Ex Taq^™^ (TaKaRa, Kyoto, Japan). Each reaction mixture (25 μL) contained 12.5 μL of 2× SYBR^®^ Premix, one μL of bovine serum albumin (25 mg mL^−1^), 0.5 μL of each primer (10 μmol L^−1^), one μL of DNA template and 9.5 μL of deionized water. These reactions were then performed in triplicate in a single run, on a plate that included a full range of the relevant standards. Standard curves were obtained using serial dilution of plasmids containing the AOA-*amoA*, AOB-*amoA*, *nirS*, *nirK* and *nosZ* genes from soil samples.

### Miseq sequencing of AOB and *nirS* containing denitrifiers communities

The AOB and *nirS* containing denitrifiers communities were analyzed with amplicon sequencing performing using Illumina Miseq platform. The bacterial *amoA* and *nirS* genes were amplified using primer amoA-1F/amoA-2R (Rotthauwe, Witzel & Liesack, 1997) and Cd3Af/R3cd (Throbäck et al., 2004), respectively. Primer amoA*-*1F and R3cd contained a unique 12 nt barcode at the 5’end for Miseq sequencing detection. The raw sequence data has been deposited on the NCBI SRA (Accession No. SRP127746). Details regarding PCR conditions and quality processing are available in the Supplemental File.

### Bioinformatics analysis

The bioinformatics analysis in our study were previously described (Yang et al., 2019a, 2019b). Specifically, raw sequences of AOB-*amoA* and *nirS* genes were processed using QIIME Pipeline Version 1.8.0 (Caporaso et al., 2010) to remove low quality (length <250 bp, with ambiguous base “N” and average base quality score <20) sequences before further analysis. Potential chimeras of AOB-*amoA* and *nirS* sequences were discarded by performing the chimera. Uchime algorithm in Mothur (Schloss et al., 2009), using RDP Fungene database (Fish et al., 2013). The remaining nonchimeric sequences of AOB-*amoA* and *nirS* were clustered into different operational taxonomic units (OTUs) using USEARCH v8.0 (Edgar, 2013) with 97% and 82% similarity level (Palmer, Biasi & Horn, 2012), respectively. Each OTU was taxonomically classified using blastn 2.2.30 against nt database, then OTUs that were not assigned as AOB and *nirS* containing denitrifiers were removed. We then constructed a neighbor joining tree using a Kimura 2-parameter distance with 1,000 bootstrap replicates in MEGA 6 (Tamura et al., 2013) to identify AOB OTUs. We used the nomenclature for AOB clusters as defined by Avrahami, Conrad & Braker (2002) and He et al. (2007). To correct the differences in the sequencing depth, the number of sequences per sample was normalized to the smallest sample size using the “sub.sample” command in the Mothur (Schloss et al., 2009).

### Statistical analysis

One-way ANOVA was used to examine the effect of compost addition on the soil ammonium and nitrate contents, gene copies of AOA-*amoA*, AOB-*amoA*, *nirS*, *nirK* and *nosZ*, OTU richness of AOB and *nirS* containing denitrifiers, relative abundance of AOB clusters at seedling, flowering and mature stage. All data above were tested for normality and homogeneity of variance before ANOVA using Levene test. Differences among treatments were then tested using a Tukey’s HSD post-hoc test at *P* < 0.05.

Permutational multivariate analysis of variance (PERMANOVA) was carried out in the vegan package (Oksanen et al., 2013) to evaluate the effects of compost addition, growth stage and their interactive effect on AOB and *nirS* containing denitrifiers community composition (Yang et al., 2019a, 2019b). Subsequently, the AOB and *nirS* containing denitrifiers community compositions were ordinated using non-metric multidimensional scaling (NMDS) with the dissimilarity matrices using the “metaMDS” function in the Vegan package (Oksanen et al., 2013). Mantel tests were applied to explore correlations between AOB and *nirS* containing denitrifiers communities and soil variables in the ecodist package (Goslee & Urban, 2007). Moreover, the “varpart” function in the vegan package was used to partition the variation of AOB and *nirS* containing denitrifiers community dissimilarity by compost addition, soybean growth stage and soil variables (SOM, TN, TP, AP, AK, pH, BD, NH_4_^+^-N and NO_3_^−^-N). Random forest analysis (Breiman, 2001) was used to explore the soil physiochemical drivers of AOA-*amoA*, AOB-*amoA*, *nirS*, *nirK* and *nosZ* gene abundance using randomForest package (Liaw & Wiener, 2002). The rfPermute package (Archer, 2016) was then used to estimate significance of importance metrics for a random forest model by permuting the response variable. All the analyses above were carried out in R (v.3.1.1) (R Core Team, 2013).

Four co-occurrence networks of soil AOB and *nirS* containing denitrifiers from CK, LC, MC and HC treatments were built using data from all three sampling times. Thus, each network was based on 12 communities, but only OTUs that occurred in at least six communities were included in the analysis. Spearman’s correlation coefficients between OTUs were calculated in each network. *P* values for multiple testing were calculated using the false discovery rate (FDR) according to Benjamini & Hochberg (1995). The Spearman’s coefficient of less than 0.6 and a *P* value of more than 0.01, were removed. The numbers of nodes and links, connectedness and modularity were calculated using the igraph package (Csárdi & Nepusz, 2006).

## Results

### Soil ammonium and nitrate content

**Figure 1:**
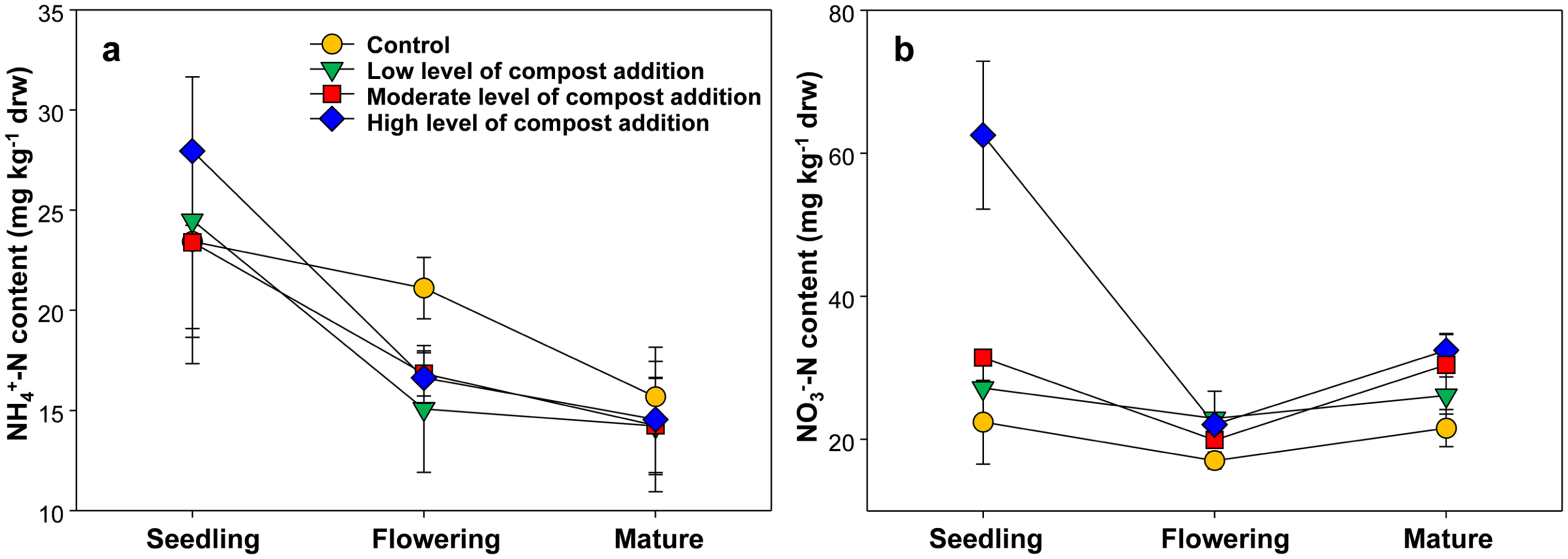
Soil NH_4_^+^-N (A) and NO_3_^−^-N (B) content among treatments in seedling, flowering and mature stage.

As shown in Fig. 1A, the soil ammonium content ranged from 14.23 ± 4.86 to 27.95 ± 7.40 mg/kg among treatments and gradually decreased across growth stages. However, soil ammonium content was unaffected by compost addition in this study (Fig. 1A; Table S2). The soil nitrate content ranged from 17.05 ± 2.15 to 62.07 ± 17.50 mg/kg and was significantly influenced by compost addition at the seedling stage (Fig. 1B; Table S2). Especially, treatment HC induced 175.6%, 129.3% and 96.2% increase in nitrate content as compared with treatment CK, LC and MC, respectively (Fig. 1B).

### Abundance of ammonia-oxidizers and denitrifiers

**Figure 2:**
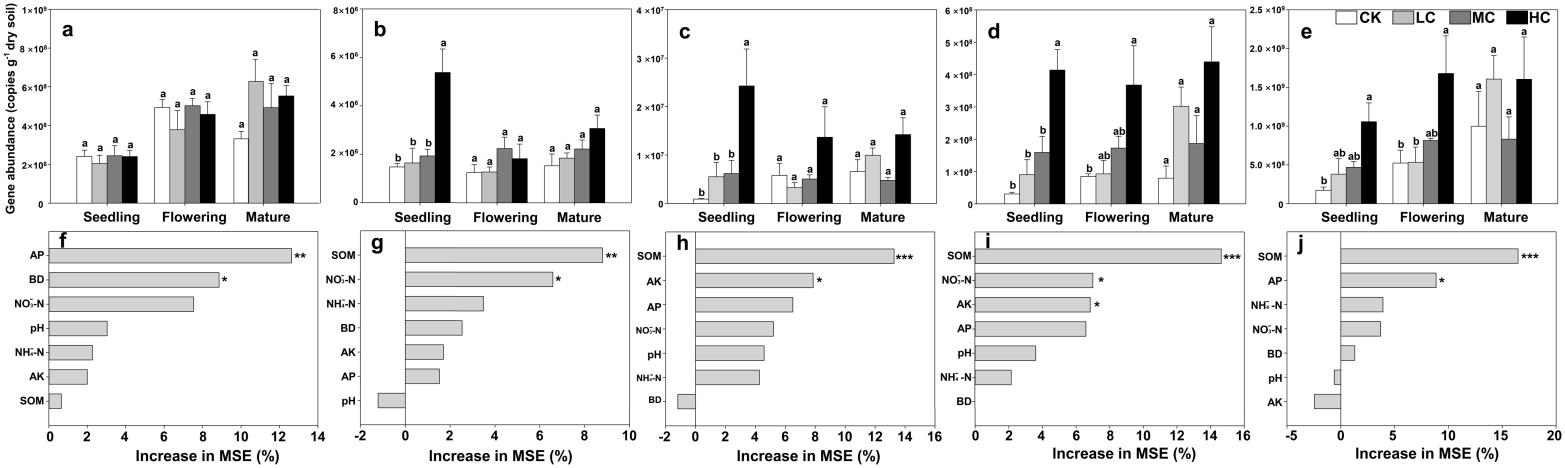
Gene copies of AOA-*amoA* (A), AOB-*amoA* (B) *nirS* (C) *nirK* (D) and *nosZ* (E); mean predictor importance of soil variables on AOA-*amoA* (F), AOB-*amoA* (G), *nirS* (H), *nirK* (I) and *nosZ* (J). Bars without shared letters indicate significant difference at *P* < 0.05. Abbreviations: CK, control; LC, low level of compost addition; MC, moderate level of compost addition; HC, high level of compost addition; BD, bulk density; SOM, soil organic matter; AP, available phosphorus; AK, available potassium; NH_4_^+^-N, ammonium; NO_3_^−^-N, nitrate; MSE, mean square error. *** *P* < 0.001; ** *P* < 0.01; * *P* < 0.05.

The copies of AOA-*amoA* gene ranged from 2.05 × 10^8^ ± 8.42 × 10^7^ to 7.18 × 10^8^ ± 2.16 × 10^7^ among treatments, and was about two orders of magnitude higher than those of AOB (Figs. 2A and 2B). However, the AOA-*amoA* gene abundance was unaffected by compost addition across the growth stages (Fig. 2A; Table S2). In contrast, one-way ANOVA analysis indicated that AOB-*amoA* gene abundance was significantly influenced by compost addition at the seedling stage (Table S2). Compared with treatment CK, treatment HC enhanced the abundance of AOB-*amoA* gene by 263% at the seedling stage (Fig. 2B). Random forest analysis showed that soil AP and BD were the major determinants of the abundance of AOA, while SOM and NO_3_^−^-N content were the major determinants of the abundance of AOB (Figs. 2F and 2G).

The gene copies of *nirS* was significantly affected by compost addition in seedling stage, while the gene copies of *nirK* and *nosZ* were significantly affected in seedling and flowering stages (Table S2). In comparison to treatment CK, soil samples from HC revealed higher abundance of *nirS*, *nirK* and *nosZ* gene abundance (Figs. 2C–2E). Random forest analysis showed that soil AP and SOM were the major determinants of *nirK* and *nosZ* gene abundance, while SOM and AK were the major determinants of *nirS* gene abundance (Figs. 2H–2J).

### Sequencing data analysis of AOB and *nirS* containing denitrifier

**Figure 3:**
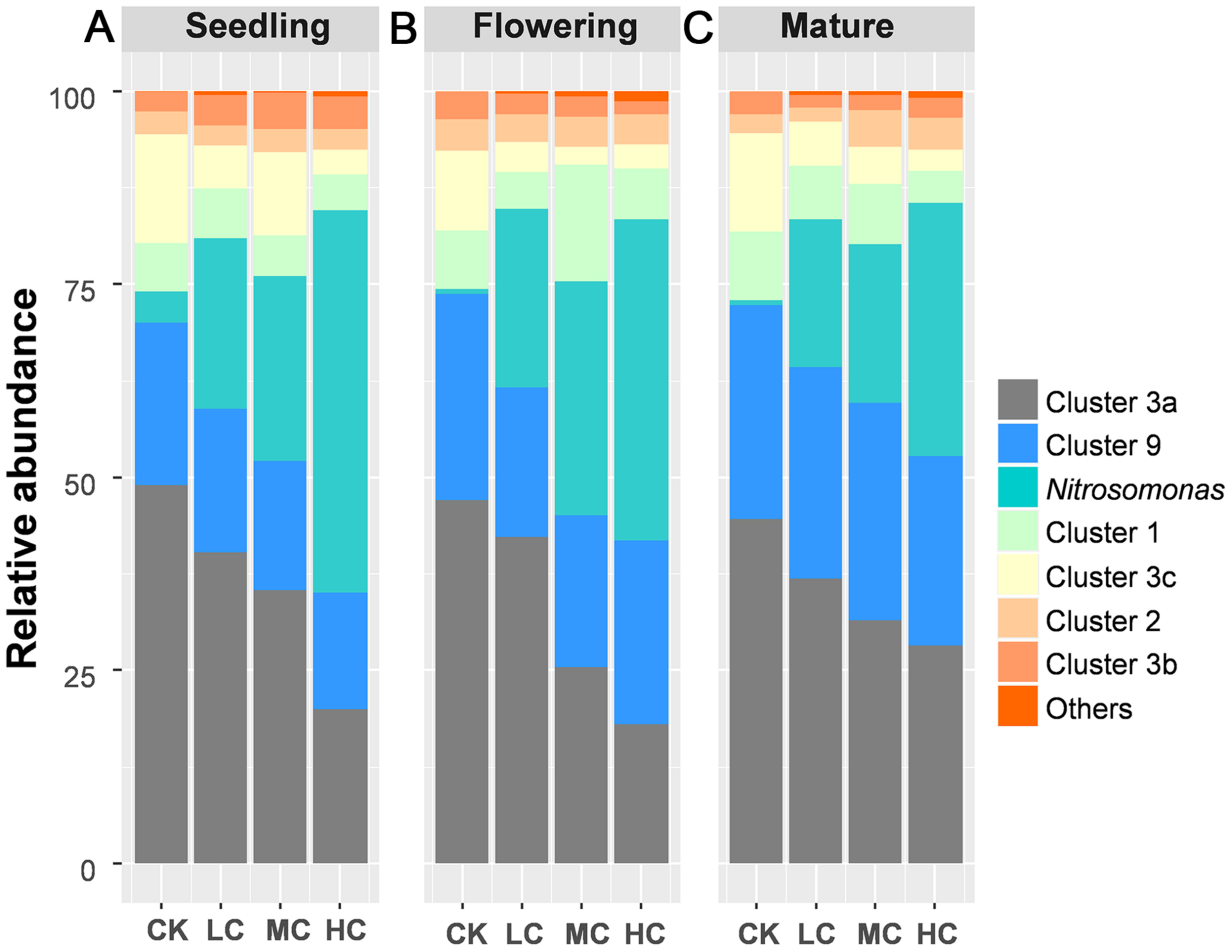
The relative abundance of the AOB lineages among treatments in seedling (A), flowering (B) and mature stage (C). Abbreviations: CK, control; LC, low level of compost addition; MC, moderate level of compost addition; HC, high level of compost addition.

A total of 883,851 AOB-*amoA* reads were obtained from 48 soil samples after quality control, from which 20,103 potential chimeras were removed. The remaining 863,748 non-chimeric reads were assigned to 62 operational taxonomic units (OTUs) based on 97% sequence similarity. The most dominant AOB OTUs were affiliated with *Nitrosospira* cluster 3a (accounting for 34.67% of the obtained AOB sequences, 13 OTUs), followed by *Nitrosomonas* (22.84%, 8 OTUs), *Nitrosospira* cluster 9 (22.27%, 13 OTUs), cluster 1 (6.98%, 1 OTU), cluster 3c (6.48%, 8 OTUs), cluster 2 (3.35%, 4 OTUs), cluster 3b (2.92%, 11 OTUs), cluster 4 (0.1%, 3 OTUs) and unclassified OTU (0.47%, 1 OTU) (Fig. S1). Among AOB lineages, *Nitrosospira* cluster 3a and *Nitrosomonas* exhibited entirely different response to compost addition. *Nitrosospira* cluster 3a, which gradually decreased along with compost application rate, was significantly decreased by HC treatment as compared with CK across the growing season (Fig. 3; Tables S3 and S4). In contrast, the relative abundance of *Nitrosomonas* was quite low in treatment CK and greatly enhanced by HC treatment (Fig. 3; Tables S3 and S4).

For *nirS* containing denitrifier, a total of 2,468,389 reads were obtained after quality control, from which 100,499 potential chimeras were removed. The remaining 2,367,890 non-chimeric reads were assigned to 98 operational taxonomic units (OTUs) based on 82% sequence similarity. The taxonomic classification of each *nirS* containing denitrifier was summarized in Table S3.

**Figure 4:**
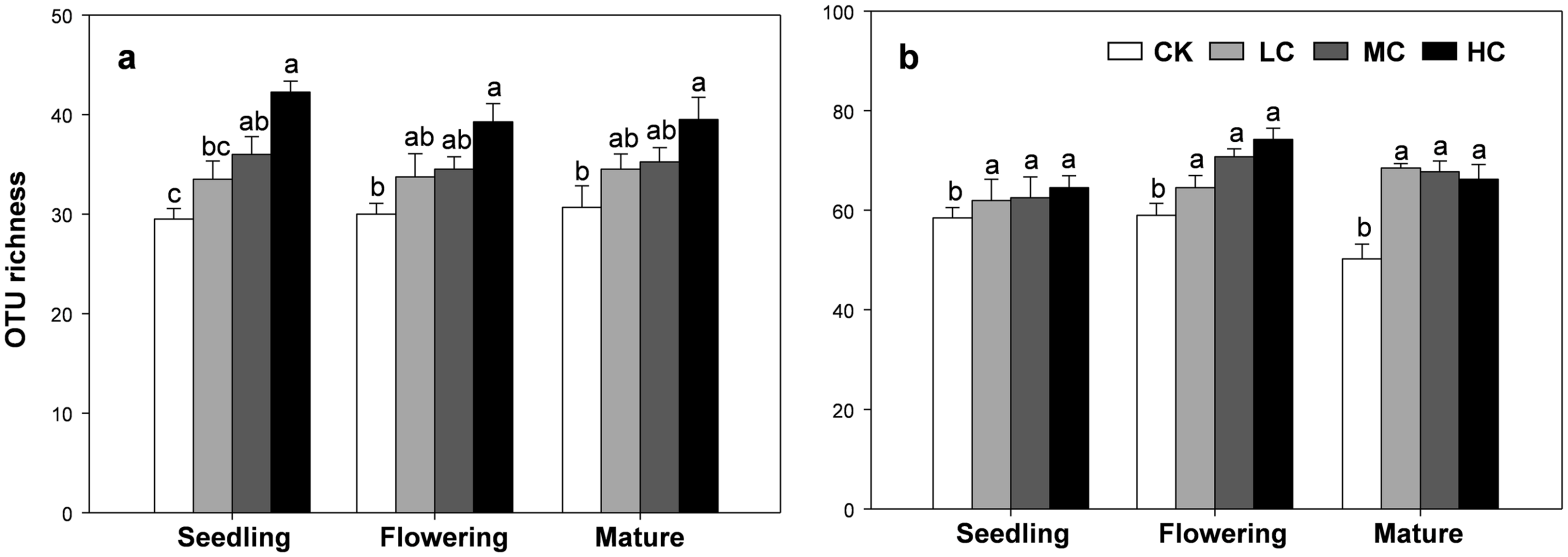
OTU richness of AOB (A) and *nirS*-containing denitrifier (B) among treatments in seedling, flowering and mature stage. Bars without shared letters indicate significant difference at *P* < 0.05. Abbreviations: CK, control; LC, low level of compost addition; MC, moderate level of compost addition; HC, high level of compost addition.

The OTU richness of AOB and *nirS* containing denitrifier were consistently influenced by compost addition across the growth stages (Table S2). Overall, the OTU richness of AOB gradually increased along with compost application rate (Fig. 4A). Compared with CK, treatment HC significantly stimulated OTU richness of AOB at all growth stages (Fig. 4A). Treatment MC, however, only induced a significant increase in AOB richness at the seedling stage, but not at flowering and mature stages (Fig. 4A). On the other hand, the OTU richness of *nirS* containing denitrifier was significantly higher in treatment LC, MC and HC than CK in all growth stages (Fig. 4B).

### Community composition of AOB and *nirS* containing denitrifier

**Figure 5:**
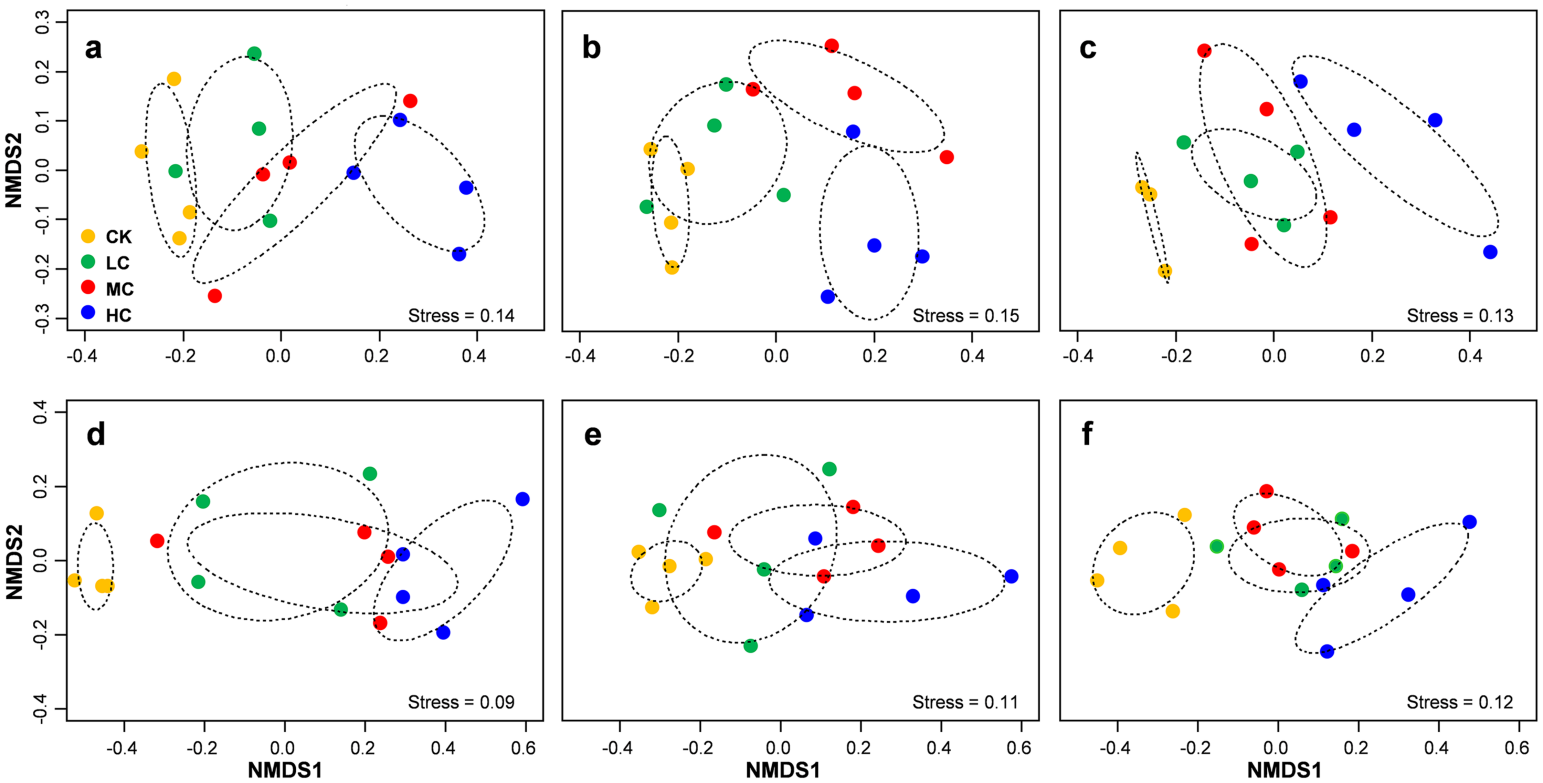
Non-metric multidimensional scaling (NMDS) of AOB community composition in seedling (A), flowering (B) and mature (C) stage; NMDS of *nirS*-containing community composition in seedling (D), flowering (E) and mature (F) stage. Circles with dashed line in NMDS plot are 95% confidence of CK, LC, MC and HC treatment. Abbreviations: CK, control; LC, low level of compost addition; MC, moderate level of compost addition; HC, high level of compost addition.

PERMANOVA analysis indicated that AOB community composition was significantly influenced by compost addition (*r*^2^ = 0.26, *P* = 0.001), marginally influenced by soybean growth stage (*r*^2^ = 0.05, *P* = 0.05) and unaffected by their interaction (*r*^2^ = 0.01, *P* = 0.62). *nirS* containing denitrifiers community composition was significantly influenced by compost addition (*r*^2^ = 0.22, *P* = 0.001), growth stage (*r*^2^ = 0.12, *P* = 0.001) and their interaction (*r*^2^ = 0.04, *P* = 0.04). Further analysis revealed that compost addition consistently influenced AOB and *nirS* containing denitrifiers community composition irrespective of growth stage (Fig. 5). Both AOB and *nirS* containing denitrifiers community compositions in treatment CK distinguished greatly from MC and HC across the growing season (Fig. 5).

Mantel tests revealed that AOB community composition was significantly correlated with pH, SOM, AP, AK and C/N, while pH, SOM and AK showed independent effect on AOB community composition (Table 1). Similarly, *nirS* containing denitrifiers community composition was significantly correlated with nitrate, SOM, AK content and C/N, while nitrate content exhibited independent effect on *nirS* containing denitrifiers community composition (Table 1). Variation partition analysis revealed that 33% of variation in AOB community composition and 35% of *nirS* containing denitrifers community compositions were explained (Fig. S2). Of these variations, 31% of AOB community was explained by soil variables, 28% by compost application rate. However, the growth stage only explained 2% of variation in AOB community. For *nirS* containing denitrifiers community, 36% of variation was explained by soil variables, 25% by compost application rate and 9% by growth stage (Fig. S2).

**Table 1:** Mantel tests and partial mantel tests of the soil ammonia oxidizer (AOB) and *nirS*-containing community with soil variables.

Soil variables	*AOB*				*nirS*			
	Mantel test		Partial mantel test		Mantel test		Partial mantel test	
	*r*	*P*	*r*	*P*	*r*	*P*	*r*	*P*
NH_4_^+^-N	−0.04	0.72	–	–	0.22	0.008	0.12	0.07
NO_3_^−^-N	0.06	0.21	–	–	0.42	0.001	0.15	0.05
BD	−0.06	0.86	–	–	0.12	0.06	–	–
SM	−0.08	0.90	–	–	0.01	0.45	–	–
pH	0.19	0.002	0.14	0.02	−0.03	0.60	–	–
SOM	0.30	0.001	0.17	0.003	0.19	0.03	0.09	0.09
AP	0.18	0.003	0.07	0.13	−0.09	0.92	–	–
TP	0.002	0.46	–	–	−0.01	0.55	–	–
AK	0.16	0.01	0.16	0.01	0.15	0.03	−0.01	0.49
TN	−0.03	0.64	–	–	−0.06	0.84	–	–
C/N	0.24	0.005	0.02	0.38	0.21	0.03	−0.03	0.67

**Note:**

BD, bulk density; SM, soil moisture; SOM, soil organic matter; AP, available phosphorus; TP, total P; AK, available potassium; TN, total nitrogen; C/N, carbon: nitrogen ratio; NH_4_^+^-N, ammonium; NO_3_^−^-N, nitrate.

### Co-occurrence networks of AOB and *nirS* containing denitrifiers

**Figure 6:**
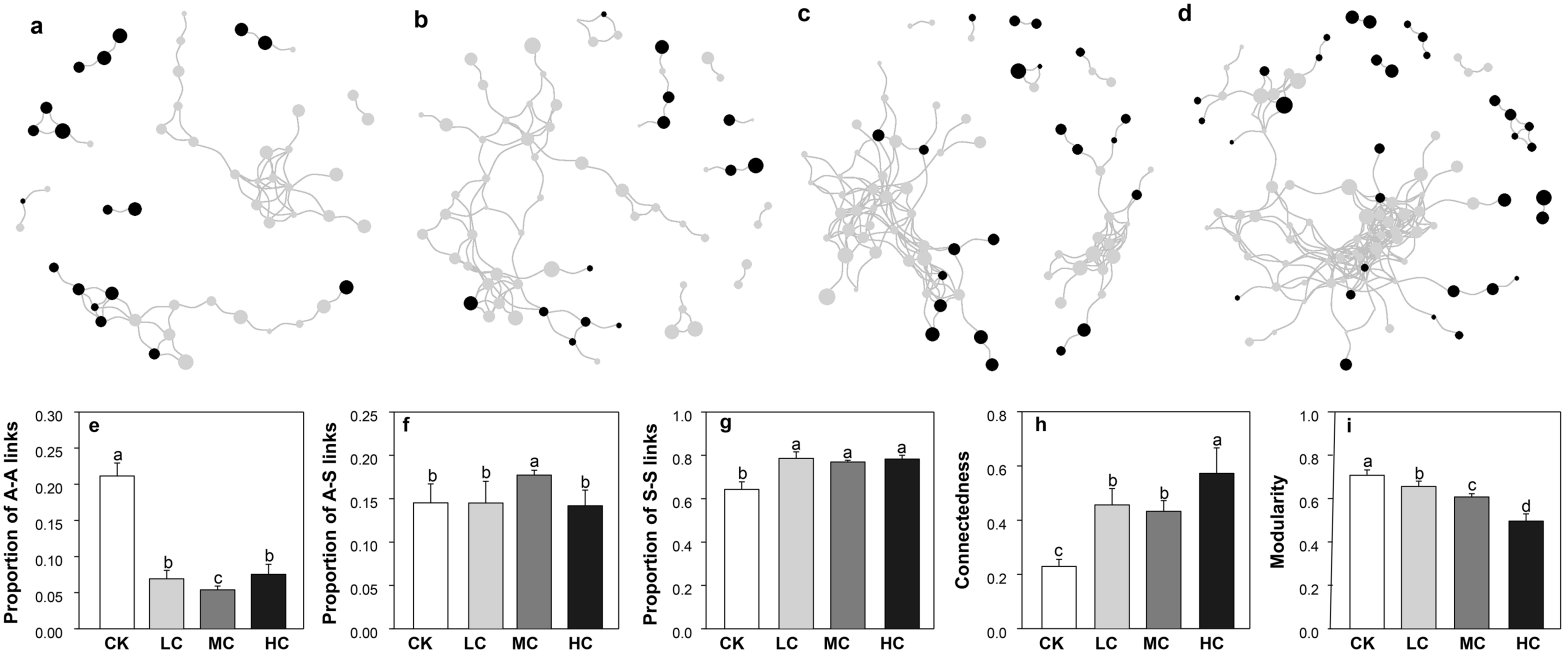
The co-occurrence networks of AOB and *nirS*-containing bacteria in CK (A), LC (B), MC (C) and HC (D) treatment; proportion of A–A links (E), proportion of A–S links (F), proportion of S–S links (G), connectedness (H) and modularity (I). Black dots represent for AOB, and grey dots represent for *nirS*-containing bacteria. The size of the circles indicates the relative abundance of each node. Abbreviations: CK, control; LC, low level of compost addition; MC, moderate level of compost addition; HC, high level of compost addition; A, AOB; S, *nirS*-containing bacteria.

The network size was smallest in CK and largest in HC, as evaluated by the number of nodes and links (Figs. 6A–6D). As shown in Fig. 6, the complexity of AOB and *nirS* containing denitrifiers network gradually increased along with the compost application rate. This pattern was also demonstrated by the network topological properties, that is, the connectedness increased along with the compost application rate, while modularity exhibited opposite trend (Figs. 6H and 6I). We also calculated the links between AOB (A) and *nirS* containing denitrifiers (S), the links between A and A and the links between S and S in each network. Notably, the compost addition greatly enhanced the proportion of S-S links (Fig. 6G), while decreased the proportion of A-A links (Fig. 6E). The proportion of A-S links was highest in treatment MC and significantly higher than others (Fig. 6F).

## Discussion

### Effect of compost addition on abundance and community of ammonia oxidizers

As organic fertilizer, compost could slowly and continuously release ammonia after ammonification (Yang et al., 2017), which may benefit the AOA and AOB growth. However, our results indicated that AOA and AOB abundance responded to compost addition in different manners. The application of compost greatly enhanced AOB abundance while having little effect on AOA across the growing season; a finding similar to this was in a cotton agroecosystem, whereby Tao et al. (2017) observed an obvious stimulating effect of manure amendment on the AOB abundance rather than AOA. It was repeatedly reported that AOA was sensitive to inorganic N-fertilizer application in low pH soil, whereas AOB was sensitive to the change of soil N availability in neutral and alkaline soils (Di et al., 2009; Schauss et al., 2009; Bi et al., 2017). For instance, organic amendment enhanced AOB abundance in neutral-pH (Liu et al., 2018) and alkaline soils (Pereg et al., 2018; Tao et al., 2017), while it enhanced AOA growth in acidic soils (Chen et al., 2011). In this study, the soil pH value was close to neutral and ranged from 6.1 to 6.6 (Table S5), which could explain why AOB rather than AOA abundance was stimulated by compost addition. In addition, compost addition could introduce exogenous microorganisms into native soil (Sun et al., 2016). In the current study, the compost indeed contained high abundances of bacterial *amoA* sequences (Table S6) as revealed by qPCR. However, given the relatively low application rate of compost (all treatments <2%) in the current study, high abundances of AOB in compost amended soils is unlikely due to the exogenous AOB introduced by compost.

Unlike soil AOB abundance, AOB community composition responded to compost amendment throughout the whole growing season. As revealed by Mantel test, both pH and SOM were key factors in shaping AOB community composition. This observation agreed with the findings of Muema, Cadisch & Rasche (2016) who reported that organic C played a vital role in regulating the community structure of ammonia oxidizing microorganisms. In addition to SOM, pH explained much of the variation in AOB community composition, which is in agreement with other studies conducted in temperate steppe (Zhang et al., 2018) and forest ecosystem (Long et al., 2012). In the current study, the pH in compost amended soils was generally higher than the control (Table S5). Therefore, soil pH may shape AOB community through direct effect on AOB growth or indirect effect on a range of soil processes (Frijlink et al., 1992).

A notable discovery was that different AOB lineages exhibited divergent response to compost addition. For instance, compost addition greatly enhanced the relative abundance of *Nitrosomonas*. As reported in previous studies, *Nitrosomonas* have often been observed in cattle manure or pig slurry amended soils (Hastings et al., 1997; Fan et al., 2011). *Nitrosospira* Cluster 3a, which was reported to be the most abundant AOB lineage in agricultural soils (Innerebner et al., 2006), gradually decreased along with compost application rate across the growing season. The different response of *Nitrosomonas* and Cluster 3a to compost addition was possibly due to their different physiological properties on ammonium. *Nitrosospira* was recognized to be the prevailing AOB in environments with low ammonium while *Nitrosomonas* is dominant in ammonium rich environments (Koops & Pommerening-Roser, 2001), which could be inversely influenced by agricultural practices. However, other clusters were unaffected by compost addition. Therefore, our results indicated that different lineages of AOB possess distinct physiological properties that could be differently influenced by agricultural practices.

### Effect of compost addition on abundance and community of denitrifiers

The abundance of denitrifier genes including *nirS*, *nirK* and *nosZ* were greatly enhanced by compost addition, which is consistent with previous studies (Kleineidam et al., 2010; Yin et al., 2015; Cui et al., 2016; Pereg et al., 2018; Tao et al., 2018). Random forest analysis indicated that SOM content contributed greatly to *nirS*, *nirK* and *nosZ* gene abundance. It was reported that most of the denitrifiers are heterotrophic (Kramer et al., 2006), therefore organic carbon might trigger their growth by providing substrates and energy (Wang et al., 2018). In addition to SOM, denitrifiers were quite sensitive to soil oxygen level (Herrmann et al., 2017). The application of compost would enhance soil microbial respiration and consume soil oxygen, creating a more suitable condition for the anaerobic denitrifiers (Attard et al., 2011; Senbayram et al., 2012).

Compost addition induced significant change in *nirS* containing denitrifiers community composition across the growing season. In the same way, Yin et al. (2015) reported that long term of manure amendment shifted *nirS* containing denitrifiers community structure in black soil. Mantel test revealed that soil NO_3_^−^*-*N content was a key factor in shaping *nirS* containing denitrifiers community composition in the current study. As the substrate of denitrification, NO_3_^−^-N strongly can strongly affect denitrification rate, thus influence *nirS* containing denitrifiers community composition (Francis et al., 2013). The shift in *nirS* community denitrifiers composition was also reflected in OTU level. For instance, OTUs that classified as *Pseudomonas*, were significantly enriched in compost amended soils (Fig. S3; Table S7). Interestingly, *Pseudomonas* took large abundance in cattle manure composting process (Maeda et al., 2010). Therefore, the *nirS* containing denitrifer existed in compost may induced shift in *nirS* containing denitrifiers community after compost addition.

### Co-occurrence networks of AOB and *nirS* containing denitrifiers

Network analysis has been increasingly used to explore the potential microbial interactions in different ecosystems (Ling et al., 2016, Yang et al., 2019b). To our knowledge, this is the first study that reports the co-occurrence network patterns of AOB and *nirS* containing denitrifiers communities. Our results indicated that compost addition significantly enhanced the complexity of A–S networks. Notably, the enhanced network complexity in compost amended soils is mainly due to the increase in S–S interactions. Higher S–S interactions under compost addition might be explained, in part, by a greater supply of organic matter, providing more opportunities for the heterotrophic *nirS* containing denitrifiers to interact with each other (Steinberger et al., 1999).

## Conclusions

In conclusion, the responses of soil ammonia oxidizers and denitrifiers were investigated across the growing season in soybean agroecosystem on the Songnen Plain. Compost addition significantly enhanced gene copies of AOB-*amoA*, *nirS*, *nirK* and *nosZ*, while AOA-*amoA* abundance was unaffected. Compost addition induced significant shift in both AOB and *nirS* containing denitrifiers community composition across the growing season. Variation of soil AOB community composition was closely correlated with soil pH, organic matter and available potassium content, while the *nirS* containing denitrifiers community was closely related to nitrate content. Network analysis indicated that the co-occurrence networks of AOB and *nirS* containing denitrifiers in compost amended soils were more complex control. Overall, our results highlighted that AOB was more sensitive to compost addition than AOA, and indicated that compost addition was a strong determinant in shaping both ammonia oxidizer and *nirS* containing denitrifier communities and co-occurrence networks in black soils.

## Supplemental Information

10.7717/peerj.8844/supp-1 Supplemental Information 1 Supplemental materials.

10.7717/peerj.8844/supp-2 Supplemental Information 2 Figure 1: Raw data of soil NH_4_^+^-N and NO_3_^−^-N content.

## Additional Information and Declarations

### Data Availability

The following information was supplied regarding data availability: All DNA sequences are available in the Sequence Read Archive (SRA): SRP127746.
