# Supplementary material for: Dynamics of ammonia oxidizers and denitrifiers in response to compost addition in black soil, Northeast China
Source: PeerJ. 2020 Apr 21;8:e8844. doi: 10.7717/peerj.8844 (PMC7182023; doi:10.7717/peerj.8844)
Supplement: Supplemental Information 1 [file peerj-08-8844-s001.docx]

**Supplementary file**

**Dynamics of ammonia oxidizers and denitrifiers in response to compost amendment in black soil, Northeast China**

Zhongzan Yang^1^,Yupeng Guan^1^, Ayodeji Bello^1^, Yanxiang Wu^1^, Jiayi Ding^1^, Leiqi Wang^1^, Yuqing Ren^1^, Guangxin Chen^1^,Wei Yang^1,*^

1. College of Resources and Environment, Northeast Agricultural University, Harbin, China

* Corresponding author

Email: yangwei_85@163.com

**PCR conditions for Miseq sequencing**

The amplification was carried out in a final 25 μL reaction solution including 2.5 μL of 10 × buffer, 1.5 mM of MgCl_2_, 200 μM of each dNTP, 0.75 μM of each primer, 0.75 U PrimeSTAR HS DNA Polymerase (Takara, Japan), and 1μL of template DNA. The AOB-*amoA* gene was amplified with the following conditions: denaturation at 94 °C for 5 min, 30 cycles of denaturation at 94 °C for 30 s, annealing at 56 °C for 30 s, and extension at 68 °C for 45 s, followed by a final extension at 72 °C for 10 min. The *nirS* gene was amplified with the following conditions: denaturation at 94 °C for 5 min, 30 cycles of denaturation at 94 °C for 30 s, annealing at 56 °C for 30 s, and extension at 68 °C for 45 s, followed by a final extension at 72 °C for 10 min. All PCR products were purified using an agarose gel DNA purification kit (TaKaRa, Japan) and quantified using Nanodrop 2000 (Thermoscientific, USA). Only PCR products with concentration > 10 ng/μL and OD 260 / OD 280≈1.8 were used, others were discarded and re-amplified to ensure the Miseq sequencing quality. The final PCR products from all samples were mixed at equimolar concentrations and then subjected to Illumina Miseq platform at Environmental Genome Platform of Chengdu Institute of Biology, Chinese Academy of Sciences.

**Table S1** Primer sets and PCR conditions used for the quantitative PCR

| Target gene | Primers | Sequence(5'–3′) | Cycling conditions | Reference |
| --- | --- | --- | --- | --- |
| AOA-*amoA* | Arch-amoAF | STAATGGTCTGGCTTAGACG | 95°C for 1 min, followed by 40 cycles of 95°C for 15s , 53°C for 45 s, 72°C for 1min. | Francis *et al*. 2005 |
|  | Arch-amoAR | GCGGCCATCCATCTGTATGT |  |  |
|  |  |  |  |  |
| AOB-*amoA* | amoA1F | GGGGTTTCTACTGGTGGT | 95°C for 1 min, followed by 40 cycles of 95°C for 15s , 55°C for 45 s, 72°C for 1min. | Rotthauwe *et al*. 1997 |
|  | amoA2R | CCCCTCKGSAAAGCCTTCTTC |  |  |
|  |  |  |  |  |
| *nirK* | nirK1F | GG(A/C)ATGGT(G/T)CC(C/G)TGGCA | 95°C for 10 min, followed by 40 cycles of 94°C for 15 s, 63°C for 45 s (6 touchdown steps to 58°C), 72°C for 45 s. | Braker *et al.* 1998 |
|  | nirK5R | GCCTCGATCAG(A/G)TT(A/G)TGG |  |  |
|  |  |  |  |  |
| *nirS* | cd3aF | GT(C/G)AACGT(C/G)AAGGA(A/G)AC(C/G)  GG | 95°C for 10 min, followed by 40 cycles of 94°C for 15 s, 62°C for 45 s (6 touchdown steps to 57°C), 72°C for 45 s. | [Throbäck *et al*. 2004](#_ENREF_12) |
|  | R3cd | GA(C/G)TTCGG(A/G)TG(C/G)GTCTTGA |  |  |
|  |  |  |  |  |
| *nosZ* | nosZ2F | CGCRACGGCAASAAGGTSMSSGT | 95°C for 10 min, followed by 40 cycles of 94°C for 15 s, 65°C for 45 s (6 touchdown steps to 60°C), 72°C for 45 s. | [Henry *et al*. 2006](#_ENREF_4) |
|  | nosZ2R | CAKRTGCAKSGCRTGGCAGAA |  |  |

| **Table S2** One way ANOVA examining the effect of compost addition (C) on the soil NH_4_^+^-N and NO3^-^-N content, gene copy numbers of ammonia oxidizers and denitrifiers (AOA-*amo*A, AOB-*amo*A, *nir*S, *nir*K and *nos*Z), and OTU richness of AOB-*amo*A and *nir*S in seedling, flowering and mature stage. | | | | | | | | |
| --- | --- | --- | --- | --- | --- | --- | --- | --- |
|  | Seedling | |  | Flowering | |  | Mature | |
|  | *F* | *P* |  | *F* | *P* |  | *F* | *P* |
| NH_4_^+^-N | 0.17 | 0.91 |  | 0.07 | 0.98 |  | 1.77 | 0.21 |
| NO_3_^-^-N | 7.90 | 0.004 |  | 1.56 | 0.25 |  | 2.48 | 0.11 |
| AOA-*amo*A | 0.22 | 0.88 |  | 0.73 | 0.56 |  | 2.82 | 0.08 |
| AOB-*amo*A | 9.67 | 0.002 |  | 1.24 | 0.34 |  | 2.43 | 0.17 |
| *nir*S | 5.73 | 0.01 |  | 1.77 | 0.21 |  | 3.33 | 0.06 |
| *nir*K | 13.21 | < 0.001 |  | 3.86 | 0.04 |  | 3.93 | 0.04 |
| *nos*Z | 5.39 | 0.01 |  | 3.97 | 0.04 |  | 0.97 | 0.44 |
| OTU richness of AOB-*amo*A | 12.84 | < 0.001 |  | 5.00 | 0.02 |  | 3.57 | 0.05 |
| OTU richness of *nir*S | 10.14 | 0.001 |  | 9.50 | .002 |  | 13.32 | < 0.001 |

| **Table S3** One way ANOVA examining the effect of compost addition (C) on *Nitrosospira* Cluster 1, Cluster 2, Cluster 4, Cluster 9, Cluster 3a, Cluster 3b, Cluster 3c and *Nitrosomonas* in seedling, flowering and mature stage. | | | | | | | | |
| --- | --- | --- | --- | --- | --- | --- | --- | --- |
|  | Seedling | |  | Flowering | |  | Mature | |
|  | *F* | *P* |  | *F* | *P* |  | *F* | *P* |
| *Nitrosospira* Cluster 1 | 0.41 | 0.75 |  | 1.45 | 0.28 |  | 0.64 | 0.60 |
| *Nitrosospira* Cluster2 | 0.02 | 0.99 |  | 0.03 | 0.99 |  | 0.88 | 0.48 |
| *Nitrosospira* Cluster4 | 0.91 | 0.46 |  | 0.80 | 0.52 |  | 1.52 | 0.27 |
| *Nitrosospira* Cluster9 | 0.52 | 0.68 |  | 0.74 | 0.55 |  | 0.24 | 0.87 |
| *Nitrosospira* Cluster3a | 7.55 | 0.004 |  | 4.46 | 0.03 |  | 1.20 | 0.35 |
| *Nitrosospira* Cluster3b | 0.38 | 0.77 |  | 2.09 | 0.16 |  | 1.32 | 0.32 |
| *Nitrosospira* Cluster3c | 1.09 | 0.39 |  | 2.78 | 0.09 |  | 3.93 | 0.04 |
| *Nitrosomonas* | 7.98 | 0.003 |  | 3.70 | .004 |  | 6.27 | 0.01 |

| **Table S4** Tukey’s HSD post-hoc test examining the Differences among treatments in seedling, flowering and mature stage. | | | | | | | | | | |
| --- | --- | --- | --- | --- | --- | --- | --- | --- | --- | --- |
| Seedling stage |  | Cluster 3a | | | |  | Nitrosomonas | | | |
|  |  | diff | lwr | upr | p adj |  | diff | lwr | upr | p adj |
|  | HC-CK | -1864.50 | -3059.72 | -669.28 | 0.00 |  | 2915.00 | 1133.01 | 4696.99 | 0.00 |
|  | LC-CK | -556.25 | -1751.47 | 638.97 | 0.53 |  | 1159.75 | -622.24 | 2941.74 | 0.27 |
|  | MC-CK | -869.25 | -2064.47 | 325.97 | 0.19 |  | 1280.75 | -501.24 | 3062.74 | 0.20 |
|  | LC-HC | 1308.25 | 113.03 | 2503.47 | 0.03 |  | -1755.25 | -3537.24 | 26.74 | 0.05 |
|  | MC-HC | 995.25 | -199.97 | 2190.47 | 0.12 |  | -1634.25 | -3416.24 | 147.74 | 0.08 |
|  | MC-LC | -313.00 | -1508.22 | 882.22 | 0.86 |  | 121.00 | -1660.99 | 1902.99 | 1.00 |
| Flowering stage |  | Cluster 3a | | | |  | Nitrosomonas | | | |
|  | HC-CK | -1870.25 | -3630.10 | -110.40 | 0.04 |  | 2628.00 | 209.50 | 5046.50 | 0.03 |
|  | LC-CK | -302.00 | -2061.85 | 1457.85 | 0.96 |  | 1441.75 | -976.75 | 3860.25 | 0.33 |
|  | MC-CK | -1391.25 | -3151.10 | 368.60 | 0.14 |  | 1895.75 | -522.75 | 4314.25 | 0.15 |
|  | LC-HC | 1568.25 | -191.60 | 3328.10 | 0.09 |  | -1186.25 | -3604.75 | 1232.25 | 0.49 |
|  | MC-HC | 479.00 | -1280.85 | 2238.85 | 0.85 |  | -732.25 | -3150.75 | 1686.25 | 0.81 |
|  | MC-LC | -1089.25 | -2849.10 | 670.60 | 0.30 |  | 454.00 | -1964.50 | 2872.50 | 0.94 |
| Mature stage |  | Cluster 3c | | | |  | Nitrosomonas | | | |
|  | HC-CK | 2069.25 | 630.56 | 3507.94 | 0.01 |  | -640.58 | -1226.97 | -54.19 | 0.03 |
|  | LC-CK | 1182.00 | -256.69 | 2620.69 | 0.12 |  | -456.58 | -1042.97 | 129.81 | 0.15 |
|  | MC-CK | 1275.00 | -163.69 | 2713.69 | 0.09 |  | -521.08 | -1107.47 | 65.31 | 0.09 |
|  | LC-HC | -887.25 | -2219.22 | 444.72 | 0.24 |  | 184.00 | -358.89 | 726.89 | 0.74 |
|  | MC-HC | -794.25 | -2126.22 | 537.72 | 0.33 |  | 119.50 | -423.39 | 662.39 | 0.91 |
|  | MC-LC | 93.00 | -1238.97 | 1424.97 | 1.00 |  | -64.50 | -607.39 | 478.39 | 0.98 |

| **Table S5** Soil bulk density (BD), soil moisture (SM), electrical conductivity (EC), pH, soil organic matter (SOM), available P (AP), available K (AK), total N (TN) and total P (TP) among treatments in the seedling stage, flowering stage and mature stage. Abbreviations: CK, no compost addition; LC, low compost addition; MC, moderate compost addition; HC, high compost addition. Values are mean (n= 4) ± SD. | | | | | | | | | | |
| --- | --- | --- | --- | --- | --- | --- | --- | --- | --- | --- |
|  |  | BD | SM | EC | pH | SOM | AP | TP | AK | TN |
| **Seedling** | CK | 1.61±0.02 | 11.64±0.55 | 63.25±11.9 | 6.15±0.13 | 20.5±1.73 | 65.21±29.79 | 0.54±0.04 | 254.67±24.58 | 1.13±0.07 |
|  | LC | 1.55±0.1 | 12.04±0.4 | 102.5±4.36 | 6.16±0.28 | 27.75±4.5 | 116.55±17.17 | 0.58±0.05 | 263.71±90.62 | 1.18±0.05 |
|  | MC | 1.6±0.1 | 11.49±0.92 | 121.5±16.94 | 6.11±0.17 | 32.72±4.7 | 109.12±25.61 | 0.6±0.03 | 314.67±41.04 | 1.27±0.06 |
|  | HC | 1.5±0.22 | 12.21±1.23 | 204±62.74 | 6.2±0.18 | 51.5±15.07 | 169.18±43.32 | 0.64±0.07 | 412.38±117.26 | 1.35±0.18 |
|  |  |  |  |  |  |  |  |  |  |  |
| **Flowering** | CK | 1.47±0.09 | 12.55±0.41 | 41.5±3.51 | 6.18±0.11 | 18.75±3.3 | 132.96±29.05 | 0.52±0.06 | 242.04±31.37 | 1.26±0.08 |
|  | LC | 1.43±0.12 | 12.09±0.75 | 58.25±12.26 | 6.26±0.24 | 24.75±4.57 | 218.48±65.38 | 0.61±0.11 | 321.33±78.31 | 1.46±0.27 |
|  | MC | 1.41±0.12 | 12.49±0.44 | 81±13.34 | 6.56±0.21 | 30.75±3.86 | 397.36±70.08 | 0.68±0.1 | 444.92±61.53 | 1.53±0.07 |
|  | HC | 1.45±0.06 | 12.84±0.83 | 85.25±13.94 | 6.48±0.19 | 41.75±4.92 | 363.33±151.97 | 0.67±0.13 | 402.13±101.44 | 1.54±0.23 |
|  |  |  |  |  |  |  |  |  |  |  |
| **Mature** | CK | 1.3±0.1 | 11.52±0.85 | 39.75±0.96 | 6.2±0.15 | 26.84±4.18 | 125.41±16.31 | 0.34±0.04 | 184.58±36.12 | 1.33±0.3 |
|  | LC | 1.36±0.05 | 11.56±1.37 | 49.25±4.5 | 6.28±0.15 | 36.86±6.21 | 237.87±34.98 | 0.4±0.07 | 210.71±31.1 | 1.43±0.21 |
|  | MC | 1.3±0.2 | 12.46±2.24 | 52±4.97 | 6.38±0.1 | 34.67±6.42 | 212.44±58.93 | 0.43±0.05 | 208.25±31.81 | 1.5±0.07 |
|  | HC | 1.35±0.05 | 11.36±1.27 | 68.75±8.54 | 6.36±0.16 | 51.5±9.16 | 358.19±76.09 | 0.64±0.14 | 283.83±52.19 | 1.57±0.31 |

| **Table S6** Gene copies of AOA-*amoA*, AOB-*amoA*, *nirS*, *nirK* and *nosZ* in compost. | | | | | |
| --- | --- | --- | --- | --- | --- |
| Genes | AOA-*amoA* | AOB-*amoA* | *nirS* | *nirK* | *nosZ* |
| Gene Copies (copies g^-1^ dry compost) | 6.88ⅹ10^7^±9.40ⅹ10^6^ | 8.24ⅹ10^6^±1.37ⅹ10^6^ | 5.25ⅹ10^7^±8.33ⅹ10^6^ | 9.12ⅹ10^7^±2.06ⅹ10^6^ | 5.79ⅹ10^7^±2.06ⅹ10^6^ |

| **Table S7** Taxonomic classification of *nirS*-denitrifier OTUs | | | |
| --- | --- | --- | --- |
| OTU name | Best BLASTN match in GenBank | Identity  (%) | Relative abundance (%) |
| nirS1 | Uncultured denitrifying bacterium | 98.289 | 15.244 |
| nirS2 | *Pseudomonas* sp. | 89.801 | 0.385 |
| nirS3 | Uncultured denitrifying bacterium | 90.191 | 2.503 |
| nirS4 | Uncultured denitrifying bacterium | 85.507 | 1.239 |
| nirS5 | Uncultured denitrifying bacterium | 99.733 | 19.668 |
| nirS6 | *Pseudomonas* balearica | 89.294 | 6.571 |
| nirS7 | Uncultured denitrifying bacterium | 92.892 | 2.718 |
| nirS8 | Uncultured denitrifying bacterium | 84.615 | 1.352 |
| nirS9 | *Pseudomonas stutzeri* | 91.101 | 8.818 |
| nirS10 | Uncultured denitrifying bacterium | 87.561 | 7.982 |
| nirS11 | Uncultured denitrifying bacterium | 85.68 | 0.731 |
| nirS12 | Uncultured denitrifying bacterium | 98.039 | 1.019 |
| nirS13 | Uncultured denitrifying bacterium | 92.771 | 1.021 |
| nirS14 | Uncultured denitrifying bacterium | 96.19 | 0.824 |
| nirS15 | Uncultured denitrifying bacterium | 95.844 | 0.756 |
| nirS16 | Uncultured denitrifying bacterium | 90.172 | 0.341 |
| nirS17 | Uncultured denitrifying bacterium | 86.508 | 1.226 |
| nirS18 | Uncultured denitrifying bacterium | 82.27 | 0.31 |
| nirS19 | Uncultured denitrifying bacterium | 93.261 | 2.65 |
| nirS20 | Uncultured denitrifying bacterium | 95.377 | 2.555 |
| nirS21 | Uncultured denitrifying bacterium | 85.819 | 0.182 |
| nirS22 | *Pseudomonas* *mandelii* | 94.161 | 1.302 |
| nirS23 | Uncultured denitrifying bacterium | 93.917 | 0.396 |
| nirS24 | Uncultured denitrifying bacterium | 100 | 0.006 |
| nirS25 | Uncultured denitrifying bacterium | 95.864 | 0.615 |
| nirS26 | Uncultured denitrifying bacterium | 94.608 | 1.061 |
| nirS27 | Uncultured denitrifying bacterium | 98.246 | 0.2 |
| nirS28 | Uncultured denitrifying bacterium | 94.217 | 2.569 |
| nirS29 | Uncultured denitrifying bacterium | 87.042 | 0.502 |
| nirS30 | Uncultured denitrifying bacterium | 98.054 | 0.26 |
| nirS31 | *Pseudomonas* *balearica* | 85.086 | 5.756 |
| nirS32 | Uncultured denitrifying bacterium | 100 | 0.028 |
| nirS33 | Uncultured denitrifying bacterium | 95.134 | 2.378 |
| nirS34 | Uncultured denitrifying bacterium | 94.349 | 0.395 |
| nirS35 | Uncultured denitrifying bacterium | 92.308 | 0.268 |
| nirS36 | Uncultured denitrifying bacterium | 91.375 | 0.133 |
| nirS37 | Uncultured denitrifying bacterium | 99.267 | 0.183 |
| nirS38 | Uncultured denitrifying bacterium | 86.842 | 0.119 |
| nirS39 | Uncultured denitrifying bacterium | 97.585 | 0.153 |
| nirS40 | Uncultured denitrifying bacterium | 94.964 | 0.335 |
| nirS41 | Uncultured denitrifying bacterium | 99.51 | 0.597 |
| nirS42 | Uncultured denitrifying bacterium | 83.693 | 0.038 |
| nirS43 | Uncultured denitrifying bacterium | 83.698 | 0.053 |
| nirS44 | Uncultured denitrifying bacterium | 86.098 | 0.04 |
| nirS45 | Uncultured denitrifying bacterium | 79.903 | 0.143 |
| nirS46 | *Pseudomonas stutzeri* | 81.752 | 0.195 |
| nirS47 | Uncultured denitrifying bacterium | 87.16 | 0.086 |
| nirS48 | Uncultured denitrifying bacterium | 98.792 | 0.223 |
| nirS49 | Uncultured denitrifying bacterium | 97.821 | 0.156 |
| nirS50 | Uncultured denitrifying bacterium | 85.956 | 0.06 |
| nirS51 | Uncultured denitrifying bacterium | 85.122 | 0.022 |
| nirS52 | Uncultured denitrifying bacterium | 88.564 | 0.348 |
| nirS53 | Uncultured denitrifying bacterium | 85.61 | 0.092 |
| nirS54 | Uncultured denitrifying bacterium | 84.45 | 0.136 |
| nirS55 | *Leptothrix cholodnii* | 89.61 | 0.032 |
| nirS56 | Uncultured denitrifying bacterium | 99.51 | 0.231 |
| nirS57 | Uncultured denitrifying bacterium | 97.297 | 0.126 |
| nirS58 | *Bacillus subtilis* | 92.683 | 0.019 |
| nirS59 | Uncultured denitrifying bacterium | 92.457 | 0.334 |
| nirS60 | *Azospirillum* sp. | 94.865 | 0.087 |
| nirS61 | Uncultured denitrifying bacterium | 77.427 | 0.024 |
| nirS62 | Uncultured denitrifying bacterium | 82.394 | 0.007 |
| nirS63 | Uncultured denitrifying bacterium | 81.034 | 0.029 |
| nirS64 | Uncultured denitrifying bacterium | 86.553 | 0.031 |
| nirS65 | Uncultured denitrifying bacterium | 81.995 | 0.002 |
| nirS66 | Uncultured denitrifying bacterium | 100 | 0.004 |
| nirS67 | Uncultured denitrifying bacterium | 81.418 | 0.009 |
| nirS68 | Uncultured denitrifying bacterium | 83.535 | 0.066 |
| nirS69 | *Azoarcus* sp. | 90.394 | 0.66 |
| nirS70 | Uncultured denitrifying bacterium | 82.767 | 0.008 |
| nirS71 | Uncultured denitrifying bacterium | 99.268 | 0.016 |
| nirS72 | Uncultured denitrifying bacterium | 99.517 | 0.124 |
| nirS73 | Uncultured denitrifying bacterium | 82.014 | 0.122 |
| nirS74 | Uncultured denitrifying bacterium | 98.575 | 0.628 |
| nirS75 | Gamma proteobacterium | 96.875 | 0.05 |
| nirS76 | Uncultured denitrifying bacterium | 97.305 | 0.063 |
| nirS77 | Uncultured denitrifying bacterium | 83.942 | 0.111 |
| nirS78 | Uncultured denitrifying bacterium | 88.509 | 0.01 |
| nirS79 | Uncultured denitrifying bacterium | 94.146 | 0.006 |
| nirS80 | Uncultured denitrifying bacterium | 88.293 | 0.026 |
| nirS81 | Uncultured denitrifying bacterium | 86.797 | 0.069 |
| nirS82 | Uncultured denitrifying bacterium | 92.892 | 0.01 |
| nirS83 | Uncultured denitrifying bacterium | 85.749 | 0.005 |
| nirS84 | Uncultured denitrifying bacterium | 83.333 | 0.005 |
| nirS85 | Uncultured denitrifying bacterium | 81.22 | 0.01 |
| nirS86 | Uncultured denitrifying bacterium | 86.553 | 0.008 |
| nirS87 | Uncultured denitrifying bacterium | 99.512 | 0.002 |
| nirS88 | *Cupriavidus* sp. | 98.025 | 0.051 |
| nirS89 | Uncultured denitrifying bacterium | 100 | 0.001 |
| nirS90 | Uncultured denitrifying bacterium | 92.892 | 0.004 |
| nirS91 | Uncultured denitrifying bacterium | 74.576 | 0.001 |
| nirS92 | Uncultured denitrifying bacterium | 95.418 | 0.036 |
| nirS93 | Uncultured denitrifying bacterium | 81.418 | 0.001 |
| nirS94 | Uncultured denitrifying bacterium | 87.651 | 0.004 |
| nirS95 | Uncultured denitrifying bacterium | 75.243 | 0.002 |
| nirS96 | Uncultured denitrifying bacterium | 91.4 | 0.007 |
| nirS97 | Uncultured denitrifying bacterium | 98.537 | 0.012 |
| nirS98 | Uncultured denitrifying bacterium | 80.147 | 0.001 |

**
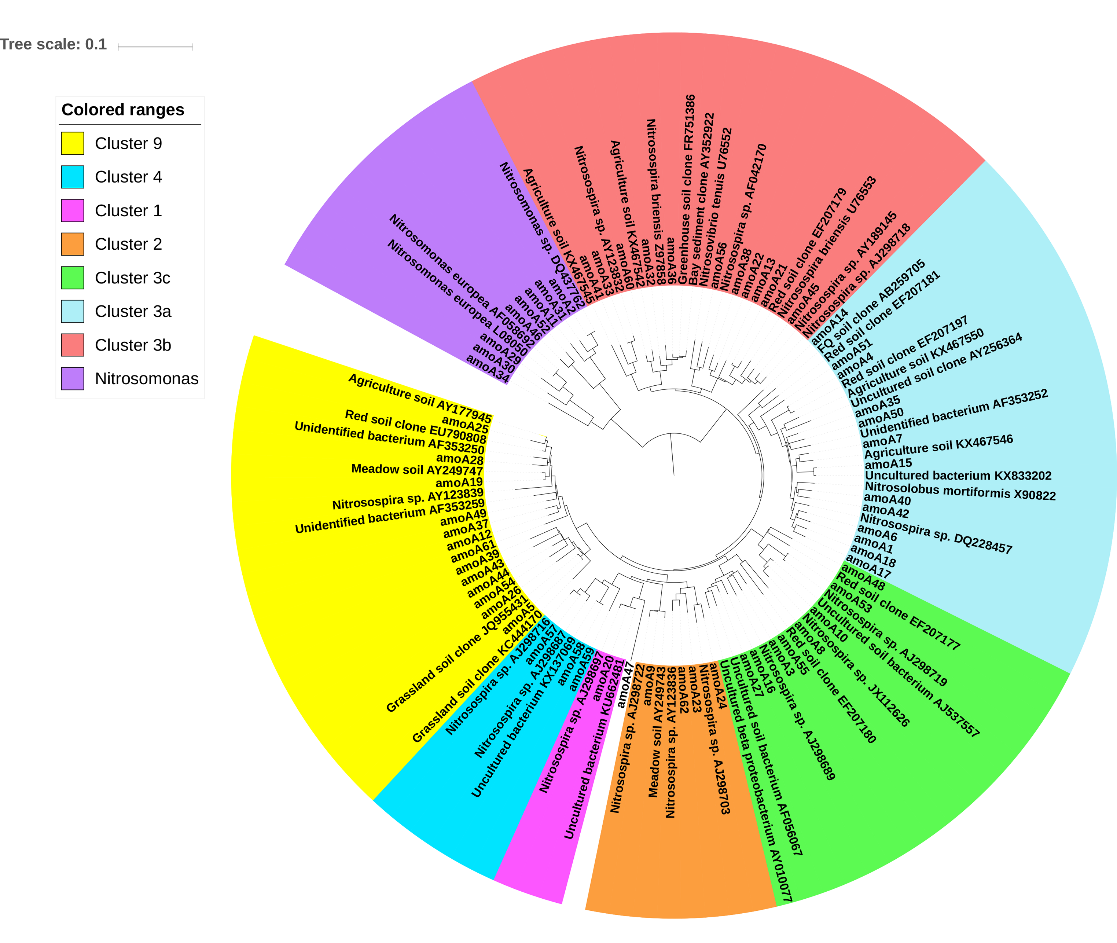
**

**Fig. S1** Neighbor-joining tree of AOB *amoA* genes. The AOB OTUs (ca. 491 bp) obtained in this study are shown with amoA in their names. References downloaded from GenBank are shown concomitantly by the corresponding accession numbers.


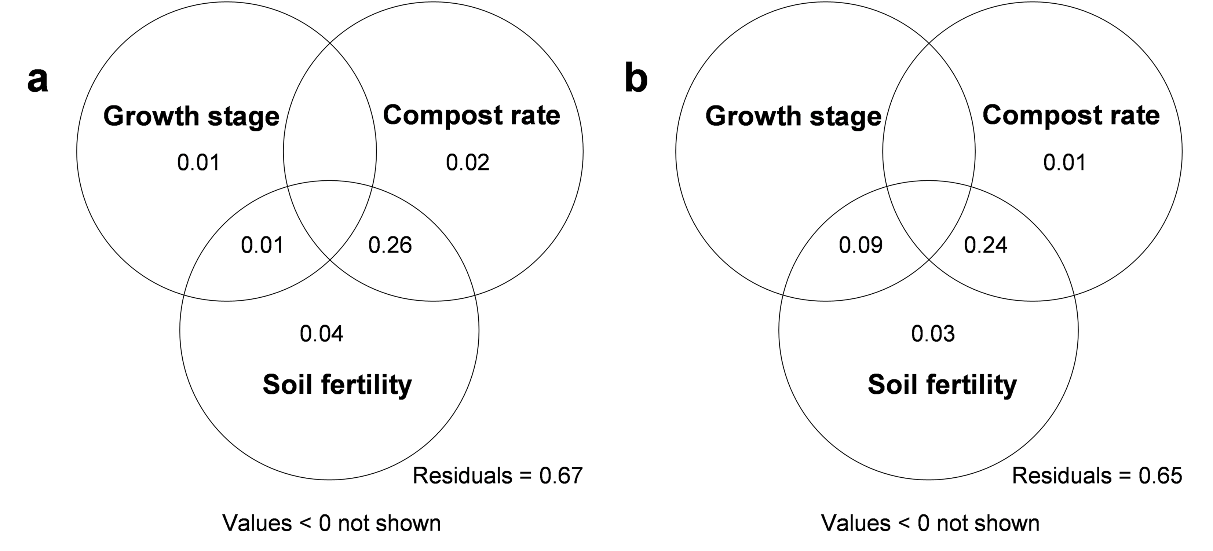


**Fig. S2** Variation partitioning analysis showing the effects of compost rate, growth stage and soil variables on AOB (a) and *nirS*-containing (b) community composition. Numbers inside circles indicate the proportion of explained variation.


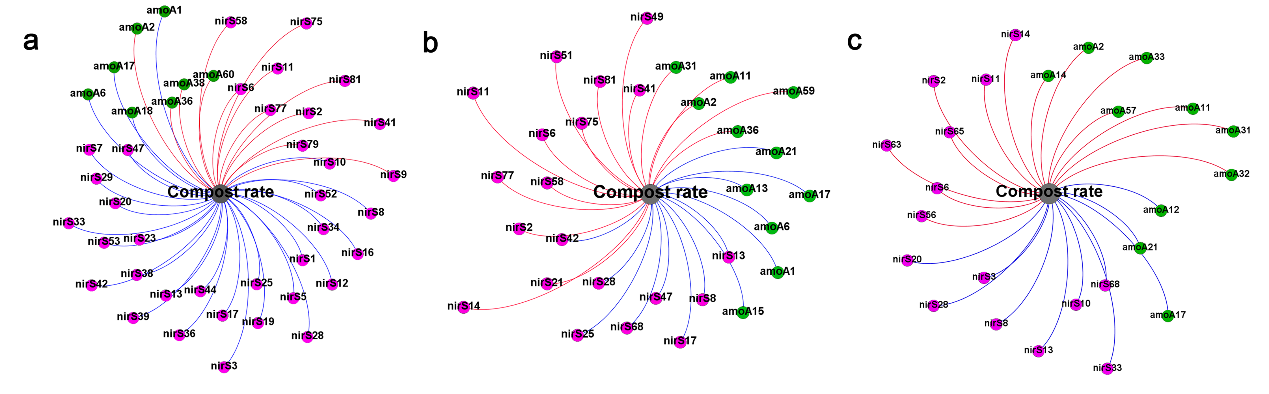


**Fig. S3** Pearson correlation analysis between the relative abundance of AOB, *nirS* OTUs and compost rate in seedling (a), flowering (b) and mature stage (c). Red lines, positive correlations; blue lines, negative correlations; green circles, AOB OTUs; pink circles, *nirS* OTUs. Line length indicates the value of correlation coefficient.

**References**

Braker G, Fesefeldt A, Witzel KP (1998) Development of PCR primer systems for amplification of nitrite reductase genes (*nirK* and *nirS*) to detect denitrifying bacteria in environmental samples. Appl Environ Microbiol 64:3769-3775

Francis CA, Roberts KJ, Beman JM, Santoro AE, Oakley BB (2005) Ubiquity and diversity of ammonia-oxidizing archaea in water columns and sediments of the ocean. Proc Natl Acad Sci USA 102:14683-14688

Henry S, Bru D, Stres B, Hallet S, Philippot L (2006) Quantitative detection of the *nosZ* gene, encoding nitrous oxide reductase, and comparison of the abundances of 16S rRNA, *narG*, *nirK*, and *nosZ* genes in soils. Appl Environ Microbiol 72:5181-5189

Rotthauwe JH, Witzel KP, Liesack W (1997) The ammonia monooxygenase structural gene *amoA* as a functional marker: Molecular fine-scale analysis of natural ammonia-oxidizing populations. Appl Environ Microbiol 63:4704-4712

Throbäck IN, Enwall K, Jarvis Å, Hallin S (2004) Reassessing PCR primers targeting *nirS*, *nirK* and *nosZ* genes for community surveys of denitrifying bacteria with DGGE. FEMS Microbiol Ecol 49:401-417
